# Absence of the *LRRK2* mutation in Emirati Parkinson’s disease patients in contrast to other Arab populations

**DOI:** 10.3389/fnagi.2025.1676115

**Published:** 2025-11-26

**Authors:** Vinod Metta, Anjana Soorajkumar, Tom Loney, Nasna Nassir, K. Ray Chaudhuri, Mohammed Jashim Uddin, Hani T. S. Benamer

**Affiliations:** 1 Department of Neurosciences, King’s College London, Institute of Psychiatry, Psychology & Neuroscience, London, United Kingdom; 2 Parkinson’s Foundation Centre of Excellence, King’s College Hospital, London, United Kingdom; 3 Kings College Hospital London, Dubai, United Arab Emirates; 4 Department of Psychiatry and Behavioral Sciences, Albert Einstein College of Medicine, New York, NY, United States; 5 Department of Basic Sciences, College of Medicine, Mohammed Bin Rashid University of Medicine and Health Sciences, Dubai Health, Dubai, United Arab Emirates; 6 Center for Applied and Translational Genomics (CATG), Mohammed Bin Rashid University of Medicine and Health Sciences, Dubai Health, Dubai, United Arab Emirates; 7 GenomeArc Inc., Mississauga, ON, Canada; 8 Department of Clinical Sciences, College of Medicine, Mohammed Bin Rashid University of Medicine and Health Sciences, Dubai Health, Dubai, United Arab Emirates

**Keywords:** Parkinson’s disease (PD), leucine-rich repeat kinase 2 (*LRRK2*), Emirati population, epidemiology, genetic

## Abstract

**Background:**

The role of genetic factors in the pathogenesis of Parkinson’s disease (PD) is characterized by heterogeneity in specific genetic variations and their prevalence across different populations and geographic locations.

**Objective:**

To investigate the frequency of the Leucine-rich repeat kinase 2 (*LRRK2*) mutation, a well-known genetic risk factor for PD, within Emirati patients.

**Methods:**

Emirati PD patients were recruited from the United Arab Emirates between September 2022 and May 2024. Blood samples were systematically screened for mutations across all 52 exons of the *LRRK2* gene.

**Results:**

The *LRRK2* mutation was not detected in any of the 50 Emirati PD patients (mean age 64.2 ± 14.1 years, of whom 56% are male) examined.

**Conclusion:**

The absence of the *LRRK2* and specifically the G2019S mutation in Emirati PD patients corroborates findings from Saudi Arabia and indicates a distinct genetic pattern compared to other Arab regions like Egypt and Maghreb (North African) countries, where the G2019S mutation prevalence ranges from 10 to 40%. This underscores the need for further research to unveil alternative genetic determinants specific to the Emirati PD population.

## Introduction

Parkinson’s disease (PD) is the second most prevalent progressive neurodegenerative disorder, primarily affecting older adults and characterized by a constellation of motor and non-motor symptoms (Ben-Shlomo et al., 2024). Despite its global impact, comprehensive investigations into the clinical and genetic characteristics of PD within Arab populations—particularly those in the Gulf Cooperation Council (GCC) countries, including Bahrain, Kuwait, Oman, Qatar, Saudi Arabia, and the United Arab Emirates (UAE)—remain limited (Benamer et al., 2008; Alamri et al., 2015). To date, the seminal study by Metta et al. represents the only in-depth analysis of both native Emirati and expatriate populations in the UAE, revealing notable endophenotypic differences (Metta et al., 2022).

Although PD is predominantly sporadic, mutations in over 20 genes have been implicated in its pathogenesis (Blauwendraat et al., 2020). Elucidating the genetic architecture of PD is essential for advancing precision medicine and developing targeted therapeutic interventions (Sardi et al., 2018; von Linstow et al., 2020). However, regional disparities—shaped by ethno-social and demographic factors—pose significant challenges to genetic research. Among the known genetic variants, the leucine-rich repeat kinase 2 (*LRRK2*) G2019S mutation is one of the most frequently reported, albeit with a markedly uneven global distribution (Simpson et al., 2022). Populations in Maghreb (North Africa) and Ashkenazi Jews exhibit the highest frequencies, with G2019S mutations accounting for 30–42% of familial and 30–39% of sporadic PD cases in the former, and up to 30 and 13%, respectively, in the latter (Lesage et al., 2006, 2008; Ishihara et al., 2007; Healy et al., 2008; Hulihan et al., 2008).

The Gulf region, like many Arab societies, is characterized by unique sociodemographic features, including large extended families and a high prevalence of consanguineous marriages. These factors increase the risk of hereditary and familial disorders (Tadmouri et al., 2009). Consequently, a higher incidence of familial PD is anticipated in these populations, underscoring the need for region-specific genetic studies. However, genetic investigations into PD within the Gulf remain scarce, with only a single study reported from Saudi Arabia (Al-Mubarak et al., 2015).

This study represents the first investigation into the frequency of *LRRK2* mutations among Emirati PD patients. By addressing this gap, it aims to contribute valuable insights into the genetic landscape of PD in the UAE and inform future research and clinical strategies tailored to this population.

## Methods

### Subject recruitment and clinical evaluation

Fifty Emirati patients diagnosed with PD were recruited between September 2022 and May 2024 from the King’s Parkinson’s Centre of Excellence and Research Centre at King’s College Hospital, Dubai, UAE. All patients were recruited from the Parkinson’s disease outpatient clinic. Diagnosis was confirmed using the UK Parkinson’s Disease Society Brain Bank criteria (Hughes et al., 1992). Written informed consent was obtained from all participants prior to enrolment. A movement disorder specialist (VM) conducted clinical evaluation and recruitment.

Demographic and clinical data collected included age at the time of assessment, age at motor symptom onset, sex, family history of PD, by asking for any immediate, first-degree family members with Idiopathic Parkinson’s disease, at least 3 generations are included, and disease stage assessed using the Hoehn and Yahr (H&Y) scale (Hoehn and Yahr, 1967), and motor symptom severity measured by the Unified Parkinson’s Disease Rating Scale Part III (UPDRS-III) (Goetz et al., 2008).

Patients were stratified according to age at onset into two categories: those with early-onset PD, defined as onset at or before 50 years of age, and those with late-onset PD, defined as onset after 50 years of age. In addition, patients were classified based on family history into three groups: familial PD, sporadic PD, defined as having no known family history of the disease; and unknown, where family history was either not reported or unavailable.

### Sample collection, DNA extraction, and whole-exome sequencing

The blood samples were collected and processed following the Standard Operating Procedure (SOP) implemented in the laboratory. Briefly, total blood was collected in EDTA tubes, snap frozen in liquid nitrogen, and stored at −80 °C until DNA was extracted. Genomic DNA was extracted from samples using the Qiagen DNA extraction Mini kit, following the manufacturer’s protocol, and quantified using a Qubit 2.0. Whole-exome sequencing (WES) was performed using the Sure Select Human All Exon V6 Enrichment Kit (Agilent Technologies, CA, USA) on an Illumina NovaSeq6000 platform (Illumina, San Diego, CA, USA). Raw fastq reads were aligned to the GRCh38 reference genome using the GATK BWA-MEM algorithm. Preprocessing steps included duplicate read removal, read group addition, and recalibration, which were conducted using GATK Base Recalibrator and Apply BQSR. Variant calling was performed using GATK Haplotype Caller and resulting VCF files were annotated using ANNOVAR and Horizon tertiary analytical platforms. Variant pathogenicity was assessed using ACMG guidelines with support from ClinVar, gnomAD, and in-silico predictors including SIFT, PolyPhen-2, and CADD. Our analysis specifically targeted pathogenic mutations across the 52 exons of the *LRRK2* gene, so we looked at any variants that might be pathogenic/damaging according to ACMG guidelines. This includes all known *LRRK2* variants and novel ones. To contextualize our findings, we screened *LRRK2* variants against whole genome sequencing (WGS) data from the Arab Pangenome Reference, comprising 53 Arab controls (negative for PD) (Nassir et al., 2025), including 42 Emirati and 11 from 7 other Arab nations.

### Ethical approval

All participants in this study provided written informed consent prior to enrolment. Ethical approval was obtained from three regulatory bodies: the Mohammed Bin Rashid University Institutional Review Board (MBRU-IRB-2022-99), the King’s College Hospital-Dubai Research Ethics Committee (KCH-REC-22/9), and the Dubai Scientific Research Ethics Committee (DSREC-07/2022–2). Approval from these institutions ensures that the study was conducted in accordance with internationally recognized ethical standards and guidelines, safeguarding the rights, dignity, and welfare of all participants.

### Statistical analysis

Statistical analyses were performed using Microsoft Excel 2019. Descriptive statistics were used to summarize the clinical characteristics of the cohort. Continuous variables were reported as mean ± standard deviation (SD), while categorical variables were expressed as frequency and percentage.

## Results

The demographic and clinical characteristics of the 50 Emirati patients with Parkinson’s disease are summarized in Table 1. Comprehensive genetic analysis revealed no pathogenic variants in the *LRRK2* gene among the participants. However, one individual was found to carry a missense variant of uncertain significance (chr12:40294857G > T; c.2821G > T; p.Asp941Tyr), located within the coding region of *LRRK2*. This variant results in the substitution of aspartic acid with tyrosine at position 941 and has not been previously classified as pathogenic in clinical variant databases. The patient with a missense variant was a 63-year-old man from Dubai with no family history or any significant past medical history, with a disease duration of 3 years, H&Y 2.5, and UPDRS-3 score of 20.

**Table 1:** The demographic and clinical profile of 50 Parkinson’s disease Emirati patients.

Characteristic	Value
Mean Age (± SD)	64.2 ± 14.1 years
Patients < 50 years	3 (Ages: 18, 22, 33)
Gender Distribution	56% Male
Mean Duration of PD from onset of symptoms (± SD)	4.9 ± 2.3 years
Mean Age of Onset (± SD)	59.5 ± 12.0 years, range 18–87 years
Early onset Parkinson’s disease (50 years or below)	8 patients (16%)
Tremor-Dominant PD	28 patients (56%)
Akinesia-Dominant PD	22 patients (44%)
Family History of PD	8% (4 patients)*
Family History Unavailable	24% (12 patients)
Mean H&Y Score (Range)	2.5 (Range: 1–5)
Mean UPDRS-3 Score (± SD, Range)	26.06 ± 7.28 (Range: 10–44)

*One had an affected father, another had an affected father, brother, and aunt, a third had a maternal aunt, and the last had a paternal uncle.

Notably, the well-established pathogenic *LRRK2* G2019S mutation, known to be prevalent in other Arab and North African populations, was not detected in any of the 50 PD Emirati patients. Similarly, no pathogenic *LRRK2* variants were identified in the Arab Pangenome Reference control dataset, which includes 42 Emirati and 11 non-Emirati Arab individuals.

## Discussion

This study represents the first investigation into the association between *LRRK2* gene mutations and Parkinson’s disease (PD) in the Emirati population. A global review reported that the *LRRK2* G2019S mutation has a prevalence exceeding 1% in 26 out of 52 surveyed countries (Simpson et al., 2022). However, our findings revealed a complete absence of the G2019S mutation among 50 Emirati PD patients. This observation is consistent with prior studies in Saudi Arabia (Al-Mubarak et al., 2015).

Although Arab populations are often grouped together based on shared language, geography, and cultural heritage across the 23 member states of the Arab League, our findings highlight important genetic distinctions within this broad classification. In contrast to the Emirati and Saudi populations, higher frequencies of the G2019S mutation have been reported in other Arab countries, including Egypt (4.38%) (William et al., 2024), and several Maghreb (North African) nations—Algeria (36%) (Lesage et al., 2006, 2008), Libya (19%) (Awayn et al., 2025) Morocco (41%) (Bouhouche et al., 2017), and Tunisia (33%) (Ishihara et al., 2007; Hulihan et al., 2008; Nishioka et al., 2010; Landoulsi et al., 2017). The higher prevalence of the G2019S mutation in Egypt may be partially explained by its geographic proximity and historical ties to the Maghreb region (William et al., 2024). Similarly, a north-to-south gradient has been observed in Europe, with lower prevalence in northern countries such as Belgium 0% (Nuytemans et al., 2008), and Denmark 1.1% (Petersen et al., 2015), and a higher prevalence in southern countries, including France (Lesage et al., 2007, 2020), Portugal (Bras et al., 2005) and Spain (Gaig et al., 2006; Gao et al., 2009; Gorostidi et al., 2009; Bandrés-Ciga et al., 2016); where prevalence hovers around 6% (Simpson et al., 2022). This pattern may reflect historical gene flow between Southern Europe and the *Maghreb*.

Interestingly, South American countries—likely influenced by Iberian ancestry—also report relatively high G2019S mutation rates, with Argentina at 3.75% (Gatto et al., 2013; Cornejo-Olivas et al., 2017), and Brazil at 2.65% (Munhoz et al., 2008; Abdalla-Carvalho et al., 2010; Chien et al., 2014; Abreu et al., 2016). In contrast, Asian populations exhibit very low prevalence, including China (0.07%), Japan (0.4%), Singapore (0%), South Korea (0%), and India (0.9%)(Simpson et al., 2022). North American populations show intermediate frequencies ranging from 1 to 2% (Simpson et al., 2022), while no cases have been reported among Black PD patients from Nigeria and Zambia (Simpson et al., 2022).

The absence of the G2019S mutation in both Saudi (Al-Mubarak et al., 2015) and Emirati PD cohorts stand in stark contrast to its high prevalence in the Maghreb, despite all being part of the Arab world. This discrepancy may be explained by the distinct ethnic compositions within these regions. The Maghreb is home to two major ethnic groups: the Amazigh (formerly known as Berbers), who are indigenous to North Africa and trace their ancestry back to the 7th century BCE, and Arabs, who migrated from the Arabian Peninsula (including current Gulf countries and Yemen) during and after the Islamic conquests of the 7th century CE (Benamer, 2008; Benamer and de Silva, 2010). The demographic transformation of the Maghreb was further shaped by the 11th-century migration of the Banu Hilal and Banu Sulaym tribes from the Arabian Peninsula (Benamer and de Silva, 2010).

A study conducted in Morocco found that 63% of individuals carrying the G2019S mutation identified as Arab, while 26% were Amazigh (Haj et al., 2017). However, the authors concluded that the *LRRK2* G2019S mutation likely originated from an Amazigh founder effect approximately 5,000 years ago (Haj et al., 2017). The high prevalence of the mutation among Arabic-speaking individuals in Morocco may reflect the extensive intermingling between Arab and Amazigh populations over centuries (Benamer and de Silva, 2010; Haj et al., 2017). The absence of the *LRRK2* G2019S mutation in populations from the Arabian Peninsula, including Saudi Arabia (Al-Mubarak et al., 2015) and the UAE (current study) may well support this hypothesis.

Taken together, these findings suggest that the distribution of the *LRRK2* G2019S mutation is shaped by complex ancestral and migratory patterns, with specific regional founder effects playing a central role. The absence of the mutation in our Emirati cohort reinforces the need for population-specific genomic studies, especially in understudied regions such as the Gulf.

Furthermore, our study utilized whole-exome sequencing to specifically screen all coding regions of the LRRK2 gene. No pathogenic or previously reported variants were identified in this cohort. Although this approach effectively captures coding variation, it does not encompass deep intronic or non-coding regulatory regions that may influence LRRK2 expression or function. In future work, we plan to extend our analysis to include other Parkinson’s disease–associated genes, as well as employ whole-genome or long-read sequencing to comprehensively investigate intronic and regulatory variants that may contribute to PD pathogenesis in the Emirati population. In conclusion, the absence of the *LRRK2* G2019S mutation in Emirati and Saudi PD patients underscores significant genetic heterogeneity within the Arab world and supports the hypothesis of an Amazigh founder effect underlying its high prevalence in the Maghreb. These findings highlight the importance of ethnically and regionally tailored genetic research in neurodegenerative diseases. However, the study’s primary limitation is its relatively small sample size of 50 patients, furthermore, the high proportion of patients with unknown family history potentially obscures the accurate classification of familial versus sporadic cases, which is a key variable in genetic studies. Despite these limitations, the clear and significant finding of the mutation’s absence provides a valuable and compelling contribution to the understanding of PD genetics across different ethnicities and geographies. Therefore, future large-scale genomic studies in Gulf populations are warranted to identify novel genetic contributors to PD and to support the development of personalized diagnostic and therapeutic strategies tailored to the unique genetic architecture of this region.

## Data Availability

Raw clinical data were generated at Kings College Hospital London, Dubai and genetic data was processed and analysed in the Center for Applied and Translational Genomics, Mohammed Bin Rashid University of Medicine and Health Sciences. Derived data supporting the findings of this study are available on request and with appropriate ethical approval and/or data sharing agreements.
